# PKM2: The Thread Linking Energy Metabolism Reprogramming with Epigenetics in Cancer

**DOI:** 10.3390/ijms150711435

**Published:** 2014-06-26

**Authors:** Ling Chen, Ying Shi, Shuang Liu, Ya Cao, Xiang Wang, Yongguang Tao

**Affiliations:** 1 Cancer Research Institute, Central South University, Changsha 410078, China; E-Mails: linglinginbeijing@gmail.com (L.C.); okoyo@163.com (Y.S.); ycao98@csu.edu.cn (Y.C.); 2 Key Laboratory of Carcinogenesis and Cancer Invasion, Ministry of Education, Changsha 410078, China; 3 Key Laboratory of Carcinogenesis, Ministry of Health, Changsha 410078, China; 4 Center for Medicine Research, Xiangya Hospital, Central South University, Changsha 410008, China; E-Mail: taoyong@csu.edu.cn; 5 Thoracic Surgery Department, 2nd Xiangya Hospital, Central South University, Changsha 410011, China

**Keywords:** PKM2 (pyruvate kinase M2), metabolic reprogramming, epigenetics, chromatin remodeling

## Abstract

Cancer metabolism reprogramming or alterations in epigenetics are linked to an incidence of cancer. It is apparent that epigenetic changes have been found in tumors, therefore, the complete epigenome and entire pathways relevant to cell metabolism are subject to epigenetic dysregulation. Here, we review the pyruvate kinase M2 (PKM2) isoform, a glycolytic enzyme involved in ATP generation and pyruvate production, which plays an essential role in tumor metabolism and growth, and also functions as a protein kinase that phosphorylates histones during genes transcription and chromatin remodeling. We also discuss the potential role of PKM2 in the dynamic integration between metabolic reprogramming and alterations in epigenetics during carcinogenesis and cancer progression.

## 1. Introduction

Cancer metabolism and epigenetics are two relatively independent and novel areas of cancer research. Recently, there has been an increasing number of studies regarding either altered tumor metabolism or altered epigenetics mechanisms in the pathogenesis or maintenance of tumors [1]. Energy metabolism, a new hallmark of cancer, is altered in many cancer cells [2]. These metabolic changes progressively impact the stable patterns of gene expression [3] or epigenetic changes [4]. Increases in isocitratedehydrogenase (IDH) mutant and the expression of small organic, acid 2-hydroxyglutarate (2-HG), inhibits the ten-eleven translocation (TET) family of enzymes, 2-oxoglutarate [5]. This product is intermediate during either passive or active DNA demethylation [6]. Moreover, the alteration of epigenetic language influences energy metabolism. Because histone-modifying enzymes are involved in several essential steps of metabolism, it is possible that they control the metabolic state of a cell through chromatin modification.

To engage in uncontrolled proliferation, cancer cells must adjust their energy acquisition by passively reprogramming their intracellular metabolism from mitochondrial respiration and oxidative phosphorylation (OXPHOS) to glycolysis and other metabolic pathways. This type of energy metabolism reprogramming, which converts glucose to lactate, disregards the accessibility of oxygen (the Warburg effect), thereby diverting carbohydrate metabolites to helpful anabolic procedures that contribute to rapid cell proliferation [7]. During the last few decades, studies of changes in cancer cell metabolism have been accompanied by studies of aerobic glycolysis and other metabolic conversions. However, the mechanism by which cancer cells meet the anabolic challenges connected with cell growth and proliferation has remained a mystery. Glycolysis converts glucose to lactate with the concomitant production of ATP. Aerobic glycolysis is the primary metabolic pathway utilized by cancer cells. Glycolytic enzymes are always greatly increased and/or deregulated in cancer cells. Pyruvate kinase (PK) is a key enzyme that determines glycolytic activity. The M2 isoform of pyruvate kinase (PKM2) controls the final and rate-limiting reaction in the glycolytic pathway. Although normal cells express the pyruvate kinase M1 isoform (PKM1), tumor cells primarily express the M2 isoform (PKM2) [8]. Switching from PKM1 to PKM2 promotes aerobic glycolysis and provides a selective advantage for tumor formation [9]. Additionally, the expression level of PKM2 is not correlated with overall survival [10]. Here, we reviewed the close link between epigenetic modifications and metabolic pathways. We hypothesize that PKM2 may play a critical role in cross-talk between the epigenetic modifications and metabolic pathways.

## 2. Pyruvate Kinase M2 may Play ACritical Role between Epigenetic Modifications and Metabolic Pathways

In the PK family, PKM2 is subject to sophisticated regulation by both oncogenes and tumor suppressors, which permits fine-tuned regulation of PKM2 function. Cancer cells present several unique metabolic phenotypes that are critical for cell growth and proliferation. Specifically, cancer cells preferentially express PKM2, which plays a key role in the Warburg effect and therefore promotes tumorigenesis [6,11]. The activity of PKM2 is negatively regulated by interaction with the CD44 adhesion molecule, a cell surface marker for cancer stem cells [12]. Therefore, this connection promotes the glycolytic phenotype of cancer cells, that are either deficient in p53 or exposed to hypoxia, and the ablation of CD44 results in marked depletion of cellular reduced glutathione (GSH) and an increased intracellular level of reactive oxygen species (ROS) in cancer cells [13]. Moreover, manipulation of the concentration of intracellular reactive oxygen species (ROS) is essential for cancer cell survival. In human lung cancer cells, acute increases in intracellular concentrations of ROS leads to inhibition of PKM2 that diverts the glucose flux into the pentose phosphate pathway, thereby generating sufficient reducing potential for the detoxification of ROS [14]. Therefore, PKM2 may play a pivotal role in balancing growth and oxidative stress. Proliferating cells nearly universally express the M2 isoform of PKM2, consequently, PKM2 is also overexpressed in nearly all tumors. In many cancers, the receptor tyrosine kinase/PI3K/AKT/mTOR pathway, an important intracellular pathway, is overactive. This decreases apoptosis and increases proliferationand alterations in cancer metabolism [15] and chromosome segregation [16]. Furthermore, this important pathway in involved in cellular energy control and glucose metabolism [6,15]. Activation of the PI3K/Akt pathway results in glucose uptake growth and glycolysis [17]. The major downstream molecule of PI3K/Akt, mTORC1, is a master regulator of cell growth and metabolism because it promotes many biosynthetic processes, including generating nucleosides and amino acids, which are required for the biosynthesis of macromolecules and organelles [15,17]. The deregulation of mTOR signaling through multistep oncogenic processes may contribute to the development of the Warburg effect in many cancers [18]. Inhibiting mTOR signaling down-regulates PKM2 expression and suppresses cancer metabolism, as demonstrated by decreased glucose uptake, lactate production (aerobic glycolysis), and reduced anabolism (macromolecule synthesis) in various cancer cell lines [18,19].

PKM2 exists as an active tetrameric form and a less active dimeric form that plays a critical role in aerobic glycolysis, whereas the former favors generation ATP via the tricarboxylic acid cycle (TAC) circle. The less active form of PKM2 promotes glucose production through aerobic glycolysis, whereas the active form of PKM2 drives glucose towards oxidative metabolism [20,21]. Fructose-1, 6-biphosphatase (FBP1) reduces PKM2 activation, and the loss of FBP1 may increase the formation of tetrameric PKM2 and glycolysis under some conditions, such as hypoxia.

Additionally, hypermethylation of the FBP1 promoter exists in basal-like breast cancer [22], indicating that there is a reverse association between PKM2 and metabolism via epigenetic changes in DNA methylation. The increase of reactive oxygen species (ROS) may decrease the active tetrameric form of cytosolic PKM2. Many metabolic enzymes are to be acetylated and their activities are modified. Acetylation of PKM2, which is dependent upon acetyl-CoA (which is derived from some of the pyruvate in mitochondria that is not converted to lactate) availability, may promote PKM2 degradation and may lead to an increased flux through anabolic synthesis pathways [6], suggesting that PKM2 acts as a glycolytic switch that can be rapidly inactivated in tumor cells by several mechanisms. Hence, a metabolic enzyme, PKM2, contribute the complex of a network that links nutrients to metabolite intermediates, epigenetic modification.

## 3. Nuclear Translocation of PKM2 (Pyruvate Kinase M2) Is Directly Linked with Cancer Metabolism

In tumor cells, PKM2 prefers to form a dimer and appears to be catalytically inactive for the reaction converting phosphoenolpyruvate (PEP) to pyruvate. As a result, it has been reported that the PKM2 dimer is an active protein kinase and the tetramer is a working pyruvate kinase. Histone H1, H3, and STAT3, are PKM2 substrate for its protein kinase activity [23]. More recently, PKM2, which is highly expressed in cancer, has been shown to be associated with suppressed mitochondrial function. As a result, activation of PKM2 in many cancers leads to reduced mitochondrial function and decrease tumor growth [24,25]. 

Under certain conditions, PKM2 translocates to the nucleus to perform its non-metabolic activities, potentially functioning as a transcriptional co-activator. During tumor progression, growth signals convert the active PKM2 form to an inactive form, therefore, the pyruvate kinase activity of PKM2 is changed and plays a “non-metabolic” role. PKM2 stimulates the transcription of various genes by interacting with and phosphorylating specific nuclear proteins, providing cancer cells with a survival and growth advantage. 

Several PKM2 binding proteins are nuclear proteins. SAICAR (succinylaminoimidazolecarboxamide ribose-5'-phosphate), an intermediate of the *de novo* purine nucleotide synthesis pathway, specifically activates PKM2. The level of SAICAR expression in cancer cells changes the cellular energy level, glucose uptake, lactate production, and promotes cancer cell survival under glucose-limited conditions through SAICAR-PKM2 interactions [26]. 

Under hypoxic conditions, the interaction of PKM2 with prolylhydroxylase 3 (PHD3) enhances PKM2 binding to hypoxia-inducible factor 1α (HIF-1α), and the PKM2 co-activator function induces glycolytic gene expression, such as the glucose transporter 1 (GLUT1), which promotes glucose uptake, lactate dehydrogenase A (LDHA), which converts pyruvate to lactate, and pyruvate dehydrogenase kinase 1 (PDK1), which inactivates pyruvate dehydrogenase, thereby, removing pyruvate from the mitochondria and decreasing O_2_ consumption [11,27], which is important for the epidermal growth factor receptor (EGFR)-induced Warburg effect.

In addition, nuclear PKM2 directly activates transcription of MEK5 by phosphorylating STAT3 at Y705 independent of the JAK2 and c-SRC pathways, which leads to the activation of transcription of STAT-targeted genes [23]. Additionally, PKCε- and NF-κB-dependent PKM2 up-regulation is involved in EGFR-promoted glycolysis and tumorigenesis, and PKM2 expression correlates with EGFR and IKKβ activity and with the grade of glioma malignancy in human glioblastoma specimens. These findings demonstrate that metabolic cooperation between the EGFR and NF-κB pathways is essential in PKM2 up-regulation and tumorigenesis [28], thereby, PKM2 is the link between growth factor signaling pathways and cancer metabolism [23,29].

## 4. Histone Acetylation Is ABridge for PKM2 between Metabolic Reprogramming and Chromatin Remodeling

Chromatin states can be inherited by the next cell generation, which preserves specific gene expression patterns, a phenomenon known as epigenetics. Epigenetics is the study of alteration in gene expression or cellular phenotype caused by relevant modifications to the genome, such as DNA methylation and histone modification, other than changes in the underlying DNA sequence [30]. Epigenetic mechanisms, including DNA methylation, RNA interference, histone variants, and posttranslational modifications, mediate chromatin structure. Recently, chromatin structure modification has been found to be closely associated with energy metabolism reprogramming.

Lysine acetylation has been shown to directly regulate energy metabolism because the majority of acetylated proteins in mitochondria are connected with various catabolic pathways. Histone acetyltransferases (HATs) are enzymes that acetylate conserved lysine amino acids on histone proteins. DNA is wrapped around histones, and by transferring an acetyl group to the histones, genes can be turned on and off. Histone acetylation increases gene expression. Because acetyl-CoA levels are important for HAT activity, it appears that these modifications are coupled to metabolism and the translation of physiological states into alterations in gene expression [2]. Histone deacetylases (HDAC) remove acetyl groups from lysines on a histone, allowing the histones wrap around the DNA much more tightly. This is important for DNA expression, which is regulated by acetylation and deacetylation. HDACs act opposite to HATs. The central function of HDACs in modulating metabolic circuits is evident in mice deficient in SIRT6, a nuclear sirtuin. Sirtuins control circadian clocks and mitochondrial biogenesis, which is activated through its association with chromatin [31]. Another finding indicates that H3K9me modifies and down-regulates genes associated with fatty acid oxidation. Therefore, histone demethylation is important for metabolic mediation. Furthermore, polycomb repressor complexes (PRCs), which are important chromatin modifiers implicated in pluripotency and cancer, regulate metabolic reprogramming. Active PRC (*PRCa*) target genes include *Hk1*, *Eno2*, *Ldha*, *Gpd1l*, and *Pck2*, which are involved in glycolysis and pyruvate metabolism [32]. This further demonstrates that histone modification is involved in metabolic reprogramming.

Protein acetylation plays an important role in the modulation of gene expression. Acetylation also occurs universally on enzymes that regulate energy metabolism, and acetylation profiles change in response to glucose availability, demonstrating that protein acetylation may be a mechanism that acts to coordinate global responses to nutrient levels. Acetyl-CoA levels have been shown to control the acetylation of histones, as well as a variety of metabolic enzymes. For PKM2, a conserved lysine residue on PKM2 (K305) is acetylated when glucose is abundant, which stimulates cancer cells to proliferate. Excess glucose increases both acetyl-CoA and acetylated PKM2 levels, which leads to PKM2 degradation. Acetylation reduces PKM2 enzymatic activity and decreases PEP affinity. Interestingly, PKM2 is acetylated by p300 acetyltransferase at K433, which is unique to PKM2 and directly contacts its allosteric activator, fructose 1,6-bisphosphate (FBP). Acetylation does not prevent PKM2 activation but promotes the nuclear accumulation and protein kinase activity of PKM2 [33]. The acetylation of PKM2 increases the complex function of PKM2 in nucleus during metabolism.

Posttranslational modifications are involved in manipulating cell functions in direct response to the levels of available nutrients [34]. Additionally, glucose is necessary for inducing the mono-ubiquitination of histone H2B at K120 (uH2B) in cells, and shRNA knockdown of PKM2 inhibited the mono-ubiquitination of histone H2B in cultured glioma cells. This novel glucose-glycolysis-uH2B signal pathway is well conserved from yeast to mammalian cells, providing an evolutionarily conserved regulatory mechanism of histone modification [35].

Additionally, PKM2 as a protein kinase directly regulates gene transcription during its non-metabolic functions of histone modification, which may be crucial for its epigenetic regulation of gene expression and tumorigenesis. It is suggested that PKM2 phosphorylates histone H1 [23]. It also has been shown that EGF uniquely regulates the subcellular distribution of PKM2 in multiple types of cancer cells. EGFR-regulated PKM2 in tumor cell promotes PKM2 binding to importin α5 and translocation to the nucleus, thereby, led to an interaction between endogenous PKM2 and β-catenin in the nucleus that is significant for β-catenin transactivation. In addition, PKM2-dependent β-catenin transactivation is required for cyclin D1 expression and activation of c-Myc transcription, resulting in the transcription of a set of Wnt/β-catenin downstream genes. This binding of β-catenin to the *CCND1* promoter region is necessary for the removal of HDAC3 from the promoter [36]. PKM2 can also directly bind to histone H3 and phosphorylates histone H3 at threonine (Thr, T) 11 depending upon EGF receptor activation, and the levels of histone H3 T11 phosphorylation correlate with nuclear PKM2 expression. This phosphorylation causes HDAC3 to be removed from the CCND1 and MYC promoter regions and acetylation of histone H3 at K9. Therefore, PKM2-regulated histone H3 modifications are important for EGF-induced expression of cyclin D1 and c-Myc, which is relevant to tumor cell proliferation, cell-cycle progression, and brain tumorigenesis.

Those findings demonstrate an additional role for PKM2 as a protein kinase. It has non-metabolic functions as a protein kinase that phosphorylates histones for gene transcription and histone modification.

Through translocation to the cell nucleus, PKM2 responds to different signals. Nuclear PKM2 participates in the regulation of gene transcription. Furthermore, growth stimulation by EGF induces PKM2 nuclear translocation and activates PKM2 for gene transcription regulation, demonstrating that the role of PKM2 in gene transcription is meditated by growth stimulation [23]. As a result, PKM2 undoubtedly has pivotal dual roles that are essential for tumor generation, the coordination of alterations in cancer cell metabolism and gene transcription that related to and required for cell proliferation. It is possible that the control of metabolism and cell proliferation by PKM2 is essential for energy metabolism reprogramming and epigenetic regulation [36].

## 5. Integration between PKM2 with Chromatin Modification

Epigenetic alterations, such as changes in the DNA methylation of the promoter regions of glycolytic enzymes, may be involved in the deregulated expression of enzymes involved in cell metabolism. Although the mechanism by which PKM2 directly regulates gene transcription remains unknown, its non-metabolic role in histone modification is essential for its epigenetic regulation of gene expression and emphasizes the cross-talkbetweenepigenomic changes and cancer.

Changes in DNA methylation are a hallmark of human cancers [1]. Cancer cells often present with universal DNA hypomethylation and hypermethylation of promoter CpG islands, causing transcriptional silencing of tumor suppressor genes and promoter genes under different stimuli [37,38,39,40].

Aberrant methylation patterns are involved in tumorigenesis, and causes genomic instability, abnormal imprinting, silencing genes and deregulated expression of oncogenes or tumor suppressor genes [41,42,43,44].The Jumonji (jmj) gene has been identified as essential roles in the development of multiple tissues in many species. However, since one of the human members of Jumonji protein has been shown to be a histone demethylase, the Jumonji C is crucial for the activation of demethylase [45]. Recently, JMJD5, a Jumonji C domain-containing dioxygenase, interacts directly with PKM2 to modulate metabolic reprogramming in cancer. The JMJD5-PKM2 interaction resides at the intersubunit interface region of PKM2, which hinders PKM2 tetramerization and blocks pyruvate kinase activity. This interaction also influences translocation of PKM2 into the nucleus and promotes hypoxia-inducible factor (HIF)-1α-mediated transactivation [46].

Long non-coding RNAs (long ncRNAs, lncRNA) are non-protein coding transcripts longer than 200 nucleotides [47]. LIN28B and its homolog LIN28A are proteins that bind to RNA and function to block the biogenesis of let-7 microRNAs [48,49]. The let-7 targets Myc, Kras, Igf2bp1 and Hmga2 are known regulators of mammalian body size and metabolism [50,51,52]. Interestingly, the target gene of let-7 overlaps those of PKM2, and it is possible that PKM2 may co-operate with let-7 to modulate metabolism.

## 6. Perspectives and Conclusions

Metabolic reprogramming in cancer has been considered as an indirect response to cell proliferation; however, recent evidence has demonstrated that metabolites themselves can be oncogenic by changing several cellular processes [6]. Epigenetic mechanisms permit an organism to respond to alterations in the environment [53]. Because these environmental changes may also occur during cell metabolism, it is possible that there is a link between cell metabolism and epigenetic modulation [54]. The co-factors associated with the methylation and acetylation of epigenetic modifications of DNA and histones arise from various metabolic pathways, including glycolysis, fatty acid oxidation, the TCA cycle, and OXPHOS.

**Figure 1:**
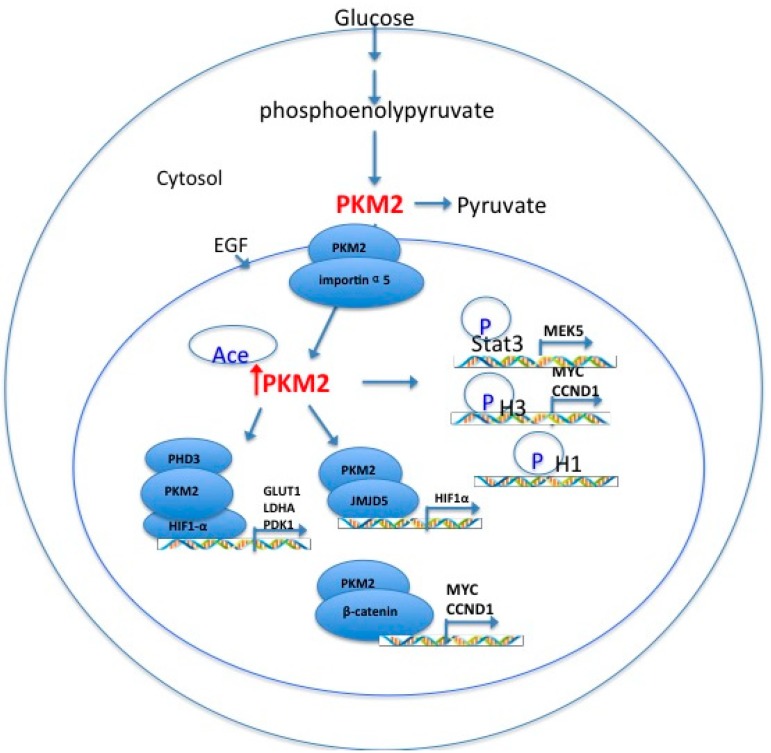
A hypothetic model illustrates the functional role of nuclear PKM2 (pyruvate kinase M2) in epigenetics and cancer cell metabolism.PKM2 can translocateinto nucleus and phosphorylate H1, H3, and STAT3 for its protein kinase activity. Furthermore, the interaction of PKM2 with prolyl hydroxylase 3 (PHD3) as well as hypoxia-inducible factor 1α (HIF1α) induces glycolytic gene expression; the intact comblex of PKM2 and β-catenin is required for cyclin D1 expression and activation of c-Myc transcription. The interaction of jumonji C domain-containing 5 (JMJD5) with PKM2 initiates HIF1α transcription by blocking its protein kinase activity.

Cancer cells express high levels of the less efficient embryonic PKM2, resulting in the inhibition of glycolysis and reduced production of ATP. PKM2 is a metabolic enzyme that can impact histone modifications. In EGFR-driven glioblastoma, PKM2 translocates to the nucleus and work as a histone kinase. Modification of the epigenetic state through alterations in metabolic enzymes is a novel phenomenon that contributes to aerobic oxidation dysfunction [2]. PKM2 also has protein tyrosine kinase activity in the nucleus, and nuclear PKM2 stimulates the transcriptional activities of HIF, β-catenin, STAT 3, and Oct4 [23,36,55]. Overall, studies of PKM2 will improve our understanding of cancer metabolism and other aspects of tumorigenesis. Future studies should determine the contributions of the cytosolic PKM2 *versus* the nuclear PKM2 dimer to aerobic glycolysis and the relationship between epigenetics and cancer cell metabolism (Figure 1). The links between epigenetics and metabolic reprogramming are only now becoming clear and are important during the very early stages of tumor progression. As we continue to explore the role of glycolytic enzymes in the regulation of chromatin, it will become easier to target cancer by resetting epigenetic abnormalities and achieving the control of cancer metabolic reprogramming.
